# Chimeric antigen receptor T-cell therapy-induced nervous system toxicity: a real-world study based on the FDA Adverse Event Reporting System database

**DOI:** 10.1186/s12885-023-11753-x

**Published:** 2024-01-02

**Authors:** Xiayang Ren, Guanmin Zhang, Guohui Li, Yanfeng Wang

**Affiliations:** 1 Department of Pharmacy, National Cancer Center, National Clinical Research Center for Cancer, Cancer Hospital, Chinese Academy of Medical Sciences and Peking Union Medical College, No. 17, Panjiayuan Nanli, Chaoyang District, Beijing, 100021 China; 2 Key Laboratory of Carcinogenesis and Translational Research (Ministry of Education/Beijing), Department of Pharmacy, Peking University Cancer Hospital & Institute, Beijing, China; 3 Department of Comprehensive Oncology, National Cancer Center, National Clinical Research Center for Cancer, Cancer Hospital, Chinese Academy of Medical Sciences and Peking Union Medical College, No. 17, Panjiayuan Nanli, Chaoyang District, Beijing, 100021 China

**Keywords:** CAR-T therapy, Nervous system toxicity, Pharmacovigilance, FDA Adverse Event Reporting System (FAERS)

## Abstract

**Background:**

Nervous system toxicity (NST) is one of the most frequent and dangerous side effects of chimeric antigen receptor T-cell (CAR-T) therapy, which is an effective treatment for related tumors in most relapsed/refractory (r/r) hematologic malignancies. Current clinical trial data do not fully reflect the real-world situation. Therefore, this study evaluated the NST of CAR-T therapy using the FDA Adverse Event Reporting System (FAERS).

**Methods:**

Data were retrieved from FAERS for the period from January 1, 2017 to March 31, 2023. Disproportionality analysis and Bayesian analysis were used for data mining. The reporting odds ratio (ROR) for NST with 95% confidence interval (CI) was calculated for each CAR-T product. The time to onset (TTO) and clinical outcomes due to CAR-T therapy-associated NST were assessed.

**Results:**

Overall, 6946 cases of NST associated with CAR-T therapy were identified. The patients had a median age of 61 years (interquartile range [IQR]: 47–69 years). Significant signals were observed for all CAR-T products (ROR: 2.19, 95% CI: 2.13–2.44). Anti-CD19 CAR-T products showed a higher NST signal than anti-B cell maturation antigen (BCMA) CAR-T products (ROR_025_ 2.13 vs. 1.98). Brexucabtagene autoleucel (ROR: 3.17, 95% CI: 2.90–3.47) and axicabtagene ciloleucel (ROR: 2.92, 95% CI: 2.81–3.03) had the two highest NST signals. For the preferred term “brain edema,” the highest signals were obtained for CD28 CAR-T products. The median TTO of NST for all CAR-T products was 7 days (IQR: 3–17 days). The proportion of death, life-threatening and hospitalization adverse events associated with NST was 20.06%, 7.21%, and 32.70%, respectively. The proportion of death outcomes was higher in patients treated with tisagenlecleucel (30.36%) than in those treated with other CAR-T products, except ciltacabtagene autoleucel (*P* < 0.001). The proportion of hospitalizations was significantly higher for lisocabtagene maraleucel-associated NST (53.85%) than for other drugs, except for ciltacabtagene autoleucel (*P* < 0.001).

**Conclusions:**

NST is more closely associated with anti-CD19 CAR-Ts and CAR-Ts containing CD28. Serious NST (brain oedema) is likely to occur with CAR-Ts that contain CD28. CAR-T-related NST warrants greater attention owing to the high proportion of serious adverse events and delayed NST.

**Supplementary Information:**

The online version contains supplementary material available at 10.1186/s12885-023-11753-x.

## Background

Chimeric antigen receptor T-cell (CAR-T) therapy has been recognized as an effective treatment for related tumors in most relapsed/refractory (r/r) hematologic malignancies [1]. Seven CAR-T therapies have been approved, including tisagenlecleucel (tisa-cel), axicabtagene ciloleucel (axi-cel), lisocabtagene maraleucel (liso-cel), brexucabtagene autoleucel (brexu-cel), and relmacabtagene autoleucel (relma-cel), which target CD-19, and idecabtagene vicleucel (ide-cel) and ciltacabtagene autoleucel (cilta-cel), which target B-cell maturation antigen (BCMA) [2–9]. These therapies have been approved by the US Food and Drug Administration (FDA), except for relma-cel, which has only been approved in China. Neurotoxicity is one of the most frequent and dangerous side effects of CAR-T therapy [10]. The American Society for Transplantation and Cellular Therapy has categorized such neurotoxicity as immune effector cell-associated neurotoxicity syndrome (ICANS) [11].

ICANS is an inflammatory neurotoxicity that is characterized by impaired blood–brain barrier integrity. The clinical manifestations of NST vary in severity and can include mild confusion and aphasia, somnolence, obtundation, and seizures and cerebral edema in more severe cases [12]. The incidence of ICANS ranges from a low of 2% to a high of 60–70% [2, 13, 14]. For example, across clinical studies of tisa-cel, axi-cel, and liso-cel for relapsed/refractory adult high-grade B-cell lymphoma, all-grade ICANS has been reported in 21% [4], 64% [2], and 25% [5] of patients, respectively, and grade 3 ICANS has been reported in 12%, 31%, and 15% of patients, respectively [2, 4, 5]. ICANS generally peaks several days after cytokine release syndrome (CRS), although rarely ICANS occurs without preceding CRS [15].

Clinical trial data, with strict entry criteria and a limited number of participants, might not fully reflect the real-world situation. A comprehensive understanding of the characteristics of CAR-T therapy-associated NST is essential to enhance approaches to prevention and management. In addition, increasing experience with CAR-T other than CD19-directed therapies can aid in addressing the questions of whether different targets are associated with different toxicity profiles, and whether ICANS is truly a single syndrome that represents a final common pathway of neuroinflammation related to immune effector cells engaging in cancer therapies to be addressed.

Therefore, we sought to assess and compare the relationship between different CAR-T therapies and NST in a large population using the US Food and Drug Administration (FDA) Adverse Event Reporting System (FAERS). We also assessed the TTO, death, life-threatening and hospitalization adverse events (AEs) for NST associated with various CAR-T products.

## Methods

### Data source

We conducted a retrospective pharmacovigilance study using the FAERS database [16]. The FAERS database collects adverse event reports from health professionals, patients, and manufacturers worldwide. These data are publicly accessible. CAR-T therapy data were retrieved from the FAERS database for the first quarter (Q1) of 2017 to (Q1) of 2023.

### Procedures

Data on NST were obtained from the REAC files according to the Medical Dictionary for Regulatory Activities (MedDRA, version 23.0) at the preferred term (PT) level based on nervous system disorders for the Standardized MedDRA Query System Organ Class 10,029,205 [17].

The CAR-T products studied included anti-CD19 cells (tisa-cel, axi-cel, liso-cel, brexu-cel) and anti-BCMA cells (ide-cell and cilta-cel). All drugs were recorded by their generic names in the DRUG file and the chosen role_code was "PS" (primary suspect) or "SS" (secondary suspect). To remove duplicate reports, the last FDA_DT was selected when the CASEID was identical and the PRIMARY_ID with the greater value was selected when the CASEID and FDA_DT were identical, as directed in the FAERS user instructions [16]. Descriptive analyses were used to synthesize the clinical features of patients with CAR-T therapy-associated NST in the data collected from the FAERS database. We calculated the TTO and NST outcomes for different CAR-T products.

### Statistical analysis

Based on the core principles of Bayesian analysis and non-proportional analysis, we used the reporting odds ratio (ROR) and the Bayesian analysis confidence propagation neural network, referred to as the information component (IC) to summarize the results. Our objective was to explore the signals associated with reported suspected AEs. The detailed equations and criteria for each algorithm are shown in Table 1. For the ROR, the signal was considered noteworthy if the lower limit of the 95% confidence interval (CI) (ROR_025_) exceeded 1, and a minimum of 3 cases were reported. For the IC, the signal was considered significant if the IC_025_ value (the lower bound of the 95% CI) exceeded zero. The presence of a significant signal indicated the potential involvement of a specific drug in the occurrence of the adverse reaction. To analyze categorical variables, we used the chi-square test for single comparisons, and the Kruskal–Wallis test for comparisons of multiple independent samples [18]. Statistical significance was set as *P* < 0.05, accompanied by 95% CIs. All statistical analyses were conducted using SPSS version 22.0 (IBM Corp., Armonk, NY, USA).

**Table 1:** Characteristics of patients with nervous system toxicity associated with CAR-T in the FAERS database (January 1, 2017 to March 31, 2023)

Characteristics	NST associated with CAR-T products	NST associated with other durgs
Total number of cases	3978	1,427,974
Patient’s age, years, median (IQR)	61 (47, 69)	59 (41, 69)
Age group, n (%)		
< 18 years	152 (3.82)	52,734 (3.69)
18–65 years	1608 (40.42)	517,237 (36.22)
≥ 65 years	1302 (32.73)	324,567 (22.73)
Unknown	916 (23.03)	533,436 (37.36)
Sex n (%)		
Male	2035 (51.16)	474,128 (33.20)
Female	1306 (32.83)	811,051 (56.80)
Unknown	637 (16.01)	142,795 (10.00)
Type of reporter, n (%)		
Health professional	2752 (69.18)	604,128 (42.31)
Non-health professional	242 (6.08)	608,285 (42.60)
Unknown	984 (24.74)	215,561 (15.09)
Reporting country, n (%)		
The United States	2707 (68.05)	869,605 (60.90)
France	184 (4.63)	61,297 (4.29)
Germany	120 (3.02)	49,762 (3.48)
Spain	119 (2.99)	15,691 (1.10)
The United Kingdom	93 (2.34)	62,066 (4.35)
Italy	85 (2.14)	30,346 (2.13)
Japan	59 (1.48)	40,575 (2.84)
Canada	48 (1.21)	88,675 (6.21)
Netherlands	42 (1.06)	10,364 (0.73)
Switzerland	39 (0.98)	3756 (0.26)
Australia	38 (0.96)	11,643 (0.82)
China	32 (0.80)	12,923 (0.91)
Other countries	99 (2.49)	132,823 (9.30)
Unknown	313 (7.87)	38,448 (2.69)
Reporting year, n (%)		
2017	1 (0.03)	204,722 (14.34)
2018	305 (7.67)	220,226 (15.42)
2019	419 (10.53)	220,436 (15.44)
2020	783 (19.68)	231,809 (16.23)
2021	839 (21.09)	206,863 (14.49)
2022	1028 (25.84)	208,369 (14.59)
2023	603 (15.16)	135,549 (9.49)

*IQR* interquartile range

The formulas were as follows:

- $${{\text{N}}}_{Expected}=\left({{\text{N}}}_{Drug}*{{\text{N}}}_{Event}\right)/{{\text{N}}}_{Total}$$
- $${\text{ROR}}=\left({{\text{N}}}_{Observed}+0.5\right)/\left({{\text{N}}}_{Expected}+0.5\right)$$
- $$IC={log}_{2}\left[\left({{\text{N}}}_{Observed}+0.5\right)/\left({{\text{N}}}_{Expected}+0.5\right)\right]$$
- $${{\text{N}}}_{Expected}\,\textrm{is the number of records expected for the target drug AE combination};$$  
- $${{\text{N}}}_{Observed}\,\textrm{is the number of observed target drug AE records};$$  
- $${{\text{N}}}_{Drug}=\textrm{is the number of any target drug}-\textrm{associated AE records};$$  
- $${{\text{N}}}_{Event}\,\textrm{is the number of target AE records};$$  
- $${{\text{N}}}_{Total}\,\textrm{is the total number of any AE records for any drug}.$$  

## Results

### Descriptive analysis

**Fig. 1:**
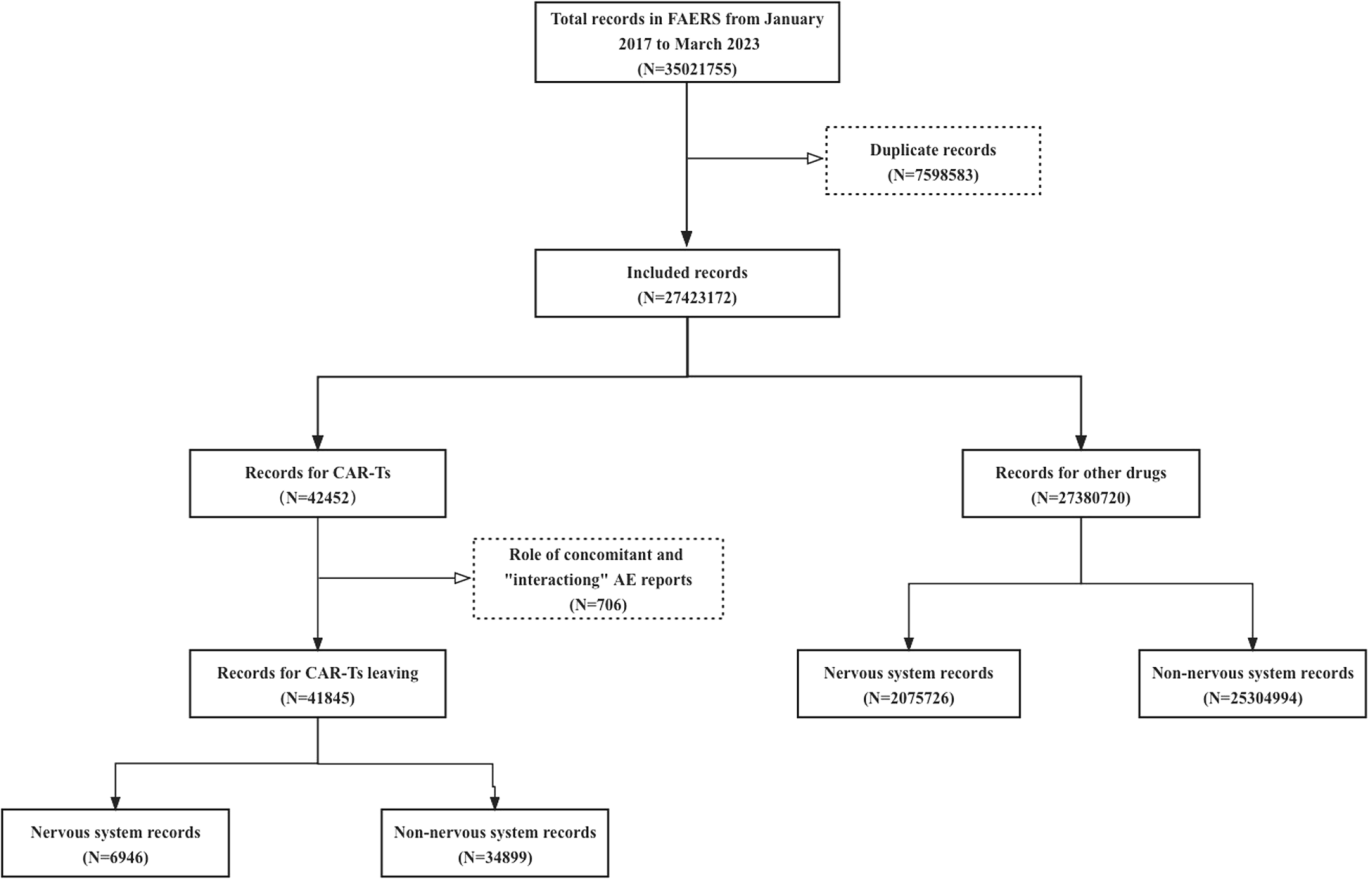
Process of selecting cases of NST associated with CAR-T therapy from the FAERS database. CAR-T, chimeric antigen receptor T-cell; FAERS, FDA Adverse Event Reporting System; NST, nervous system toxicity

From January 2017 to March 2023, a total of 35,031,755 reports were extracted from the FAERS database, and 27,423,172 reports were included in the final analysis; of them 42,452 reports on CAR-T and 6,946 cases of NST associated with CAR-T as the suspected drug were identified, as shown in Fig. 1. The clinical features of the cases are summarized in Table 1. The median patient age was 61 years (IQR: 47–69 years). The proportion of male patients (51.16%) was higher than that of female patients (32.83%). Most reports of NST with CAR-T were submitted by health professionals (69.18%), and were reported from the United States (68.05%). Furthermore, the number of reports increased steadily over time.

### Signal values associated with CAR-T therapy

The signal values of NST and ICANS associated with CAR-T are shown in Table 2 and Table S1 respectively. In general, the ROR and IC associated with CAR-T therapy were statistically significant. Overall, CAR-T therapy was associated with a significant excess in reports of NST (ROR: 2.19, 95% CI: 2.13–2.24, IC_025_: 1.09). A majority of NST associated with CAR-T were reported for axi-cel (*n* = 3659, 52.68%), with a relatively strong signal (ROR: 2.92, 95% CI: 2.81–3.03, IC_025_: 1.49). Tisa-cel was the second most frequently reported CAR-T product (*n* = 2097, 30.19%), but it had the lowest signal values (ROR: 1.41, 95% CI: 1.34–1.47, IC_025_: 0.42). The number of reports was higher for anti-CD19 CAR-T products than for anti-BCMA CAR-T products (*n* = 6562, 94.47%; vs. *n* = 384, 5.53%), but the NST signal value for anti-CD19 CAR-T products (ROR: 2.18, 95% CI: 2.13–2.24, IC_025_: 1.09) was only slightly higher than that for anti-BCMA CAR-T products (ROR: 2.21, 95% CI: 1.98–2.46, IC_025_: 0.97). Anti-CD19 CAR-T (ROR: 455.42, 95% CI: 399.29–519.44) has stronger ICANS signals than anti-BCMA product (ROR: 124.58, 95% CI: 98.66–157.30). Axicabtagene ciloleucel and Brexucabtagene autoleucel have the strongest two ICANS signals (Axicabtagene ciloleucel ROR: 526.91, 95% CI: 475.03–584.44; Brexucabtagene autoleucel ROR: 285.19, 95% CI: 242.96–334.76).

**Table 2:**
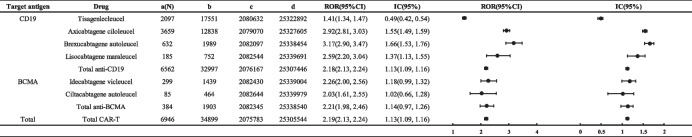
Associations of NST with different CAR-T products

A, number of reports containing both the suspect drug and NST; b, number of reports containing both the suspect drug and all other adverse events (except NST); c: number of reports containing both other medications (except the drug of interest) and NST; d: number of reports containing other medications (except the drug of interest) and all other adverse events (except NST). *NST* nervous system toxicity, *CAR-T* Chimeric Antigen Receptor-T cell, *ROR* reporting odds ratio, *IC* information component, *95% CI* the 95% two-sided confidence interval

## The Signal spectrum of NST in CAR-T therapy

**Fig. 2:**
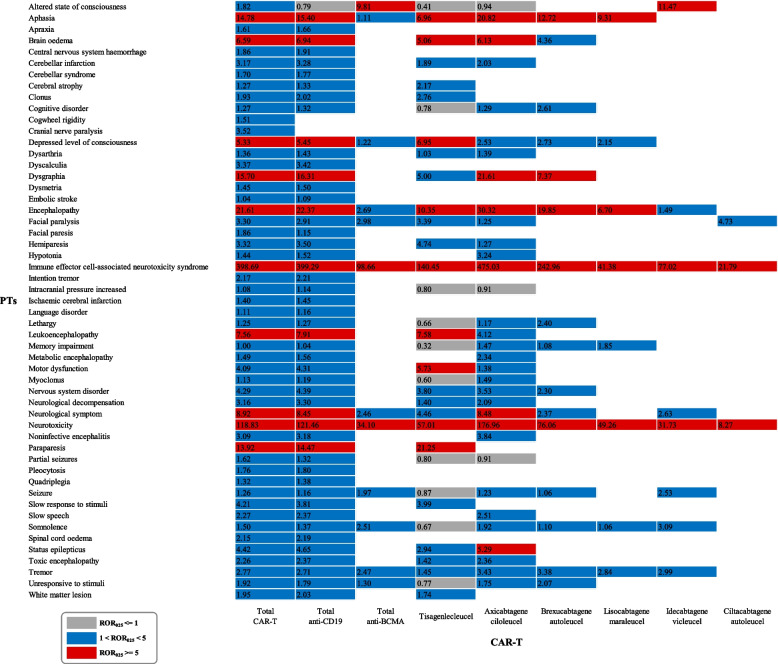
Neurotoxicity Signal Profiles of Different CAR-T products

The NST signal was detected in different CAR-T therapies (Fig. 2). The top 10 most frequently reported NST associated with CAR-T therapy were neurotoxicity (*n* = 1769, 25.47%), ICANS (*n* = 1266, 18.23%), encephalopathy (*n* = 375, 5.40%), headache (*n* = 364, 5.24%), aphasia (*n* = 317, 4.56%), tremor (*n* = 312, 4.49%), somnolence (*n* = 219, 3.15%), depressed level of consciousness (*n* = 159, 2.29%), seizure (*n* = 147, 2.12%), and memory impairment (*n* = 116, 1.67%). Despite the detection of many disproportionality signals, the text covered only the top 3 most significant disproportionality signals (based on the ROR_025_ value) for each CAR-T product. For tisa-cel, 27 disproportionality signals were significant, including ICANS (ROR_025_: 140.45), neurotoxicity (ROR_025_: 57.01), and paraparesis (ROR_025_: 21.25). For axi-cel, 33 disproportionality signals were significant, including ICANS (ROR_025_: 475.03), neurotoxicity (ROR_025_: 176.96), and encephalopathy (ROR_025_: 30.32). For brexu-cel, 16 disproportionality signals were significant, including ICANS (ROR_025_: 242.96), neurotoxicity (ROR_025_: 76.06), and encephalopathy (ROR_025_: 19.85). For liso-cel, only eight disproportionality signals were significant, including neurotoxicity (ROR_025_: 49.26), ICANS (ROR_025_: 41.38), and aphasia (ROR_025_: 9.31). For ide-cel, 11 disproportionality signals were significant, including ICANS (ROR_025_: 77.02), neurotoxicity (ROR_025_: 31.73), and altered state of consciousness (ROR_025_: 11.47). Finally, for cilta-cel, only three disproportionality signals were significant, including ICANS (ROR_025_: 21.79), neurotoxicity (ROR_025_: 8.27), and facial paralysis (ROR_025_: 4.73). Our study showed a high signal value for brain edema in all CAR-T therapies (ROR_025_ = 6.59), including anti-CD19 CAR-T therapies (tisa-cel ROR_025_ = 5.06, axi-cel ROR_025_ = 6.13, and brexu-cel ROR_025_ = 4.36).

## Time to onset of CAR-T therapy-associated NST

**Fig. 3:**
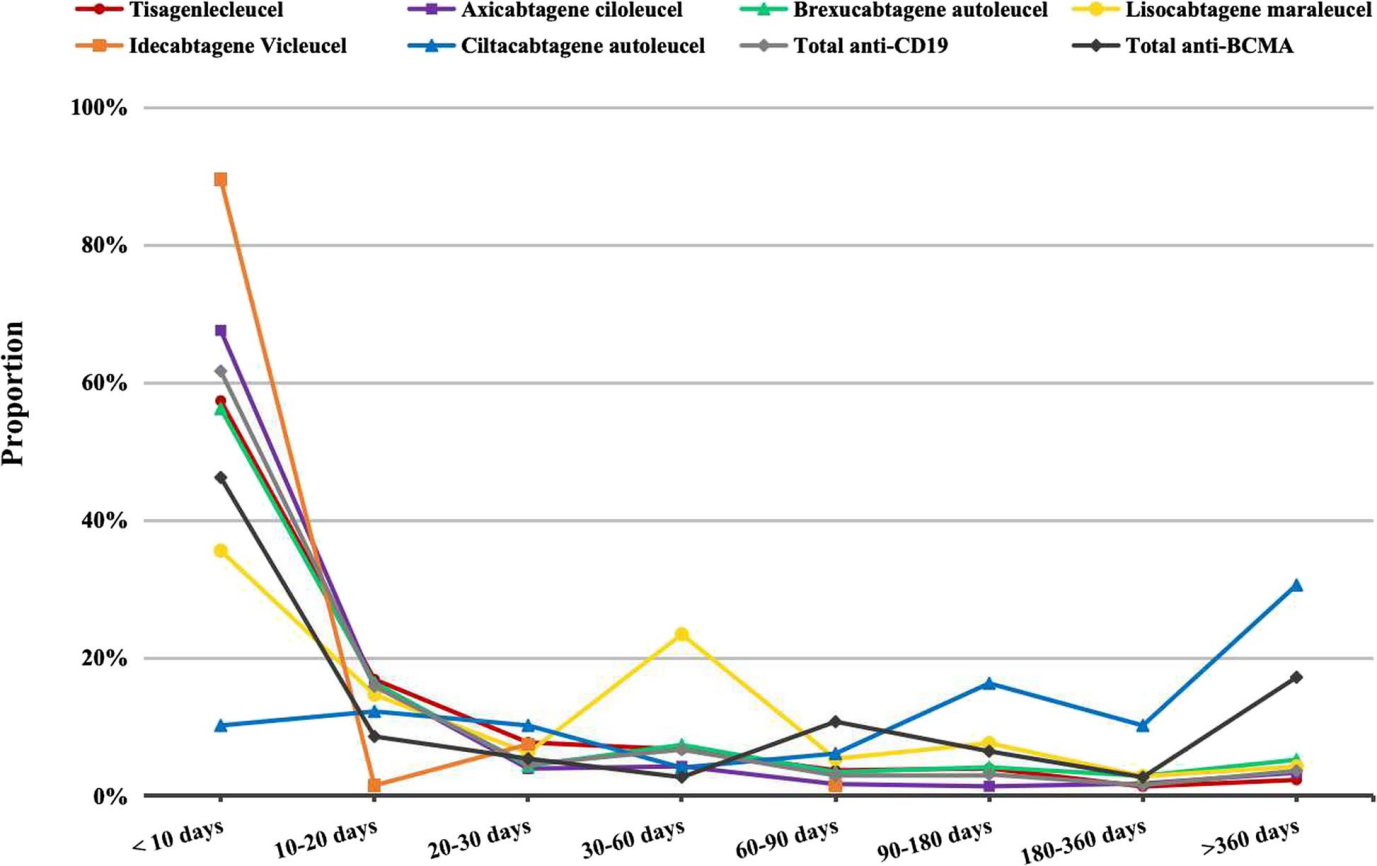
Time to onset of adverse effects of NST from CAR-T

The TTO of NST and ICANS for each CAR-T product is shown in Fig. 3 and Table S2 in the attachment. The median TTO of NST was 8 days (IQR: 3–20 days) for tisa-cel, 6 days (IQR: 2–25 days) for axi-cel, 8 days (IQR: 4–25 days) for brexu-cel, 19 days (IQR: 6–53 days) for liso-cel, 2 days (IQR: 1–7 days) for ide-cel, and 120 days (IQR: 20–433 days) for cilta-cel. The median TTO of NST for anti-CD19 and anti-BCMA CAR-Ts were 7 days (IQR: 3–23 days) and 14 days (IQR: 3–103 days) respectively. The median TTO of NST and ICANS for all CAR-T products was 7 days (IQR: 3–17 days) and 4 days (IQR: 2–11 days) respectively. NST developed within 10 days in most cases, except for cases associated with cilta-cel therapy.

### Death, life-threatening AEs, and hospitalization due to NST associated with CAR-T

**Fig. 4:**
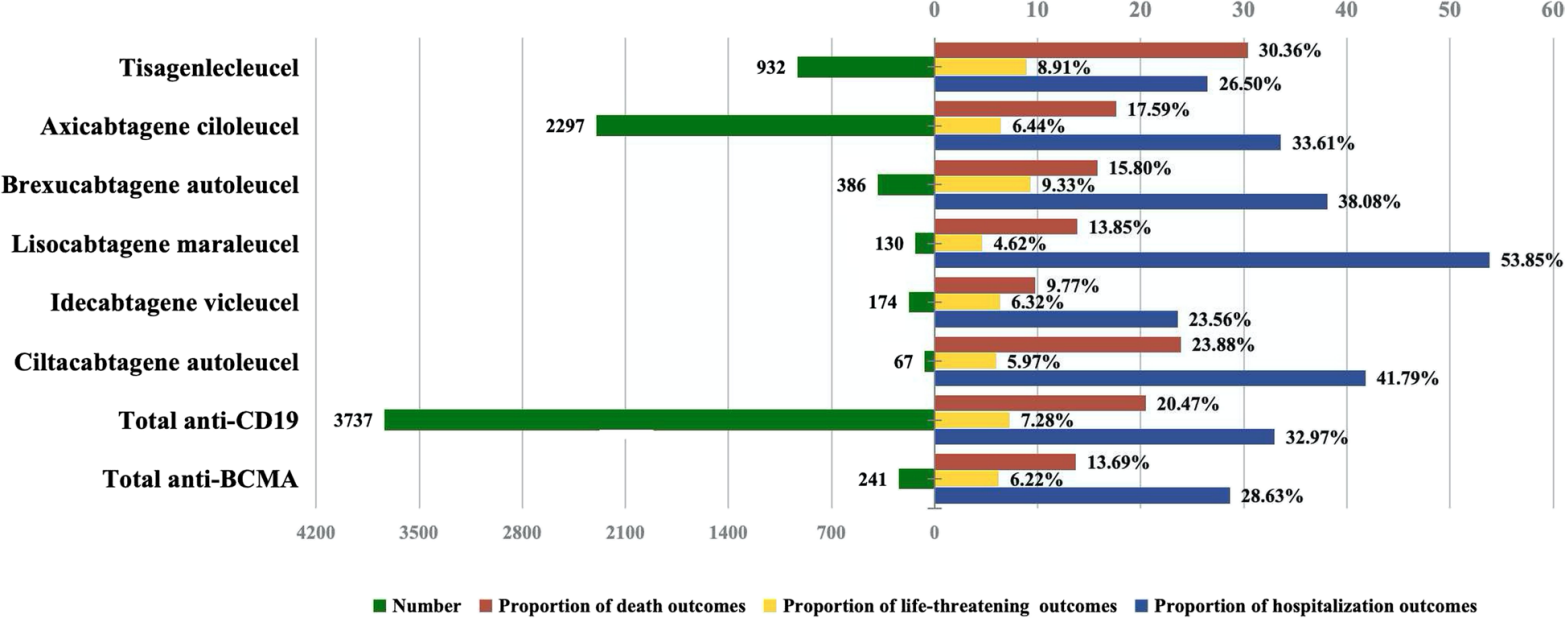
Proportion of death, life-threatening AEs and hospitalization for CAR-T-associated NST

To analyze the prognosis of CAR-T-associated NST, we assessed the proportion of death, life-threatening AEs, and hospitalization due to NST following therapy with different CAR-T products. The results are shown in Fig. 4 and Table S3 in the attachment. Overall, CAR-T-associated NST resulted in death, life-threatening AEs, and hospitalization in 20.06%, 7.21%, and 32.70%, of reported cases, respectively. The proportion of death among patients with NST associated with tisa-cel (30.36%) was significantly higher than that reported with other CAR-T products, except for cilta-cel (*P* < 0.001 for group comparison, chi-square test). The proportion of life-threatening AEs did not differ significantly by product, (*P* = 0.078 for group comparison, Fisher’s exact test). Patients with liso-cel-associated NST had a higher hospitalization outcome rate (53.85%) than for other CAR-T products, except for cilta-cel (*P* < 0.001 for group comparison, chi-square test). Anti-CD19 CAR-T had a higher proportion of death than anti-BCMA CAR-T (20.47% vs. 13.69%, *P* = 0.011, chi-square test).

## Discussion

Our study is the most extensive compilation of the incidence, clinical characteristics, and outcomes of NST associated with specific CAR-T products in real-world settings conducted to date. Our assessment was based on data sourced from the FAERS pharmacovigilance database. This study demonstrated an association between CAR-T therapy and NST, and revealed the characteristics associated with different CAR-T products.

The pathogenesis of ICANS is not completely understood, but it appears to be related to cytokine-induced toxicity rather than a non-tumor effect on the target [12]. Neurologic AEs typically occur after the onset of CRS, and ICANS often develops in the setting of improving or resolved CRS. Although ICANS is usually preceded by CRS, ICANS does not occur in all patients with severe CRS, and not all patients with ICANS exhibit preceding CRS. The blood–brain barrier is disrupted, and endothelial cells are activated by a cascade of endothelial cell activation and systemic inflammation after CAR T-cell infusion, leading to hemorrhage and cerebral edema in severe cases [19–22]. Serum levels of proinflammatory cytokines, including interleukin 2 (IL-2) and IL-15, and cytokines associated with endothelial cell activation, such as IL-6, interferon gamma (IFN-γ), and tumor necrosis factor alpha (TNF-α), were higher in patients who developed severe ICANS [23].

Owing to the earlier approval of anti-CD19 CAR-T than anti-BCMA CAR-T, a majority of reports were of patients treated with anti-CD19 CAR-T. In this study, the signal of anti-CD19 CAR-T was only slightly higher than that of anti-BCMA CAR-T. The incidence of ICANS appears to be higher for CD19-targeting CAR-T therapies than for anti-BCMA CAR-T, possibly due to the robust T-cell expansion observed with anti-CD19 CAR-T products [23]. Neurological toxicity is reported less frequently in studies of anti-BCMA CAR-T therapy than in those for anti-CD19 CAR-T, but owing to the small number of published studies on anti-BCMA CAR-T, this finding is inconclusive [24].

Second-generation CARs, which are composed of CD3 and a costimulatory domain (CD28 or 4-1BB [CD137]), are the basis of all currently marketed CAR-T products. The intracellular domains of axi-cel and brexu-cel consist of CD3ζ and the CD28 costimulatory domain. The intracellular domain for other CAR-T therapies consist of CD3ζ and the 4-1BB costimulatory domain [1].

It is probable that high-grade ICANS is more common with CART-T products that have CD28 as a costimulatory domain, as ICANs is reported in up to 45% of patients treated [2, 25–27]. This difference was initially attributed to the faster expansion of the CAR T with CD28 than with the 4-1BB costimulatory domains [12]. Conversely, for 4-1BB-containing CAR-T, the frequency of ICANS is lower, with severe ICANS being reported in 13% of patients using tisa-cel [3, 28]. Our study revealed similar results that CAR-Ts containing CD28 produced the two highest signal values.

In this study, encephalopathy and aphasia were the clinical manifestations with the highest signal values for most CAR-T products, which differs from a previous study that reported that transient cognitive impairment was the most common clinical manifestation of ICANS [19]. Aphasia, especially anomia, and word-finding defects could be a valuable early warning sign of ICANS [23]. The most serious neurological complication is cerebral edema, which is fatal in most cases. Despite the lack of comprehensive data, based on the reported incidence across clinical trials, we estimated that 1–2% of patients treated with CD19-CAR-T develop cerebral edema [19]. Some anti-CD19 CAR-T products have been reported to be associated with fatal cerebral edema, but cerebral edema appears to be extremely uncommon with tisa-cel and axi-cel [29]. The reported incidence of fatal neurotoxicity due to cerebral edema associated with anti-CD19 CAR-T treatment with either CD28 or 4-1BB constructs is 3% [22, 27]. In all cases of fatal cerebral edema, CRS was a factor, and severe CRS is associated with severe ICANS [2, 3, 21, 22, 30–32]. Our study showed a high signal value for brain edema in all CAR-T therapies.

Language disturbance, which is one of the common manifestations of ICANS, may have a different mechanism, compared with that of the more dangerous manifestations, such as cerebral edema; however, no consistent distinguishing parameters have reported to explain why ICANS manifests differently in different patients [21].

Seizures, including clinical convulsions and electrographic seizures without a motor correlate, have been reported in 5–10% of patients who receive CAR-T therapy, with an incidence of 0–30% with CD28-co-stimulated anti-CD19 CAR-T therapy, and 3–14% with 4-1BB anti-CD19 CAR-T therapy [21]. In our study, anti-BCMA CAR-T products showed higher signal than did anti-CD19 CAR-T products in the “seizure” PT (ROR_025_ 1.97 vs. ROR_025_ 1.16). All CAR-T products received significant signals in the “tremor” PT in our study. Although tremor is not considered a core sign of ICANS for diagnostic purposes, it frequently co-occurs with or precedes more definitive neurological impairment [21].

The time course of ICANS is usually monophasic, with clinical manifestations quickly rising to their peak, and then improving over time [25]. Typically, the onset of ICANS occurs after the peak CRS severity [12]. In clinical trials, the median TTO of ICANS has been reported to be 4–5 days after infusion. The clinical manifestations generally last for 5–10 days [12] and resolution typically occurs within 3–8 weeks [2, 3, 22, 24]. A pooled analysis found a mean TTO of ICANS of 6.4 ± 3.2 days and mean duration of 8.3 ± 10.5 days [33]. In this study, NST had a longer TTO, with a median of 7 days for all CAR-T products. The onset of NST occurred earlier in patients treated with CD28 co-stimulated CAR-T cells than in those treated with products containing the 4-1BB co-stimulatory domain. A previous study reported a median TTO of ICANS of 4 days for axi-cel, and 6 days for tisa-cel [23]. Our research has shown different results that the median TTO of 5 days for axi-cel and 4 days for tisa-cel. ICANS was defined as any new and well-defined neurological symptom that occurred within 60 days of infusion of CAR-T cells and was attributed to infusion [32]. In most patients, ICANS resolves within 3 weeks, although prolonged encephalopathy lasting up to 173 days duration has been reported [23]. Long-term follow-up studies of patients treated with CAR-T are ongoing, and late-onset neurological events have been reported [23]. In this study, the duration of NST varied according to the CAR-T product, and was longest for cilta-cel, with which 30.61% of cases lasted more than 360 days.

Although the course of ICANS is often mild and time limited, some patients develop severe and potentially fatal ICANS or experience long-term neuropsychiatric effects that persist for more than 1 year after treatment [21, 22, 34–38]. ICANS can occur without antecedent CRS, but tends to be mild in such cases. Most cases of ICANS resolve within 4 weeks of onset [12]. Severe clinical manifestations of ICANS are generally associated with early onset of CRS, possibly due to a high dose of CAR-T or an unusually robust and rapid growth of CAR T cells [39]. The manifestations of ICANS can develop rapidly in cases with concomitant cerebral edema, in which patients can progress from being neurologically normal to dying from brain herniation within 24 h [19]. Although reversible in most cases, ICANS can prolong hospitalization, require intensive care, delay recovery, and increase the cost of care [40]. In patients who are identified as being at high risk of developing ICANS, inpatient treatment, earlier imaging, or more frequent monitoring may be necessary [23]. Long-term follow-up of patients is necessary due to the possibility of delayed neurotoxicity.

Our study has few limitations. First, FAERS is a spontaneous reporting system (SRS). It does not allow for direct safety comparisons, quantification of associations, or calculation of incidence rates owing to missing denominator data [41]. The data collected is not capable of quantifying the adverse reaction signals based on the total number of adverse reactions. The signal intensity between drugs and reactions was only used to provide a qualitative indicator. Second, SRS data are generally less reliable than those gathered in clinical trials and cohort studies. The identification and reporting of AEs within the SRS are subject to less stringent control.

Third, it is difficult to identify significant risk factors between CAR-Ts and NST, since the deficiency of pre-existing nervous system disorders and comorbidities that may have impacts on the nervous system. Additionally, this study was not limited to a specific disease or dosage, which is significantly different from clinical trials. Fourth, Anti-CD19 CAR-T products accounted for most of the reported data, and only 5.53% of the reported cases were received anti-BCMA CAR-T. There may be considerable bias due to underreporting. Fifth, the calculation, justification and power analysis of the sample size selected in this study were not performed, because all eligible adverse drug reactions will be included. Finally, data mining revealed imperfect reporting with inaccuracies and incomplete entries, potentially causing analytical bias. Despite the limitations of FAERS, our findings shed light on essential CAR-T-associated NST aspects, and provide a basis for rigorous research to confirm the results.

## Conclusion

Based on the FAERS database, we profiled NST associated with different CAR-T products and provided details on the occurrences, clinical features, and prognosis. NST is more closely associated with anti-CD19 CAR-Ts and CAR-Ts containing CD28. Serious NST (brain oedema) is likely to occur with CAR-Ts that contain CD28. Owing to the high proportion of serious AEs and delayed NST, more attention should be paid to CAR-T-related NST.

### Supplementary Information

**Additional file 1:** **Table S1.** Associations of ICANS with different CAR-T therapies. **Table S2.** TTO of ICANS with different CAR-T therapies. **Table S3.** Clinical outcome proportions of ICANS with different CAR-T therapies.

## Data Availability

The data that support the findings of this study are available upon from the corresponding author, Yanfeng Wang, on reasonable request. The raw data can be obtained from the FAERS database at the following link: FAERS Quarterly Data Extract Files (fda.gov).

## Abbreviations

AE: Adverse event
axi-cel: Axicabtagene ciloleucel
BCMA: B cell maturation antigen
brexu-cel: Brexucabtagene autoleucel
CAR: Chimeric antigen receptor
CAR-T: Chimeric antigen receptor T cell
CI: Confidence interval
cil-cel: Ciltacabtagene autoleucel
CRS: Cytokine release syndrome
FAERS: FDA Adverse Event Reporting System
FDA: US Food and Drug Administration
IC: Information component
IC_025_: Lower 95% confidence limit of the IC
ICANS: Immune effector cell-associated neurotoxicity syndrome
ide-cel: Idecabtagene vicleucel
IQR: Interquartile range
liso-cel: Lisocabtagene maraleucel
NST: Nervous system toxicity
PT: Preferred term
relma-cel: Relmacabtagene autoleucel
ROR: Reporting odds ratio
ROR_025_: Lower 95% confidence limit of the ROR
tisa-cel: Tisagenlecleucel
TTO: Time to onset
