# Supplementary material for: Chimeric antigen receptor T-cell therapy-induced nervous system toxicity: a real-world study based on the FDA Adverse Event Reporting System database
Source: BMC Cancer. 2024 Jan 2;24:10. doi: 10.1186/s12885-023-11753-x (PMC10762809; doi:10.1186/s12885-023-11753-x)
Supplement: Supplementary file 1 — Additional file 1: Table S1. Associations of ICANS with different CAR-T therapies. Table S2. TTO of ICANS with different CAR-T therapies. Table S3. Clinical outcome proportions of ICANS with different CAR-T therapies. [file 12885_2023_11753_MOESM1_ESM.docx]

**Supplemental file**

**Table S1. Associations of ICANS with different CAR-T therapies**

**Table S2. TTO of ICANS with different CAR-T therapies**

**Table S3. Clinical outcome proportions of ICANS with different CAR-T therapies**

**Table S1.** **Associations of ICANS with different CAR-T therapies**

| Target antigen | Drug | a(N) | b | c | d | ROR (95%CI) | IC (95%CI) |
| --- | --- | --- | --- | --- | --- | --- | --- |
| CD19 | Tisagenlecleucel | 249 | 19399 | 1215 | 27402309 | 161.08(140.45,184.74) | 7.33(7.12,7.48) |
|  | Axicabtagene ciloleucel | 727 | 15770 | 737 | 27405938 | 526.91(475.03,584.44) | 9.04(8.92,9.13) |
|  | Brexucabtagene autoleucel | 182 | 2439 | 1282 | 27419269 | 285.19(242.96,334.76) | 8.16(7.91,8.33) |
|  | Lisocabtagene maraleucel | 32 | 905 | 1432 | 27420803 | 59.09(41.38,84.38) | 5.88(5.30,6.30) |
| Total anti-CD19 | | 1189 | 38370 | 275 | 27383338 | 455.42(399.29,519.44) | 8.83(8.74,8.90) |
| BCMA | Idecabtagene vicleucel | 59 | 1679 | 1405 | 27420029 | 100.37(77.02,130.81) | 6.65(6.22,6.96) |
|  | Ciltacabtagene autoleucel | 18 | 531 | 1446 | 27421177 | 34.95(21.79,56.07) | 5.13(4.33,5.68) |
| Total anti- BCMA | | 77 | 2210 | 1387 | 27419498 | 124.58(98.66,157.30) | 6.96(6.58,7.23) |
| Total CAR-Ts | | 1266 | 40579 | 198 | 27381129 | 463.25(398.69,538.28) | 8.86(8.76,8.92) |

**Table S2.** **TTO of ICANS with different CAR-T therapies**

|  | Tisagenlecleucel | Axicabtagene ciloleucel | Brexucabtagene autoleucel | Lisocabtagene maraleucel | Idecabtagene vicleucel | Ciltacabtagene autoleucel | Anti-CD19 CAR-T | Anti-BCMA CAR-T | Total CAR-Ts |
| --- | --- | --- | --- | --- | --- | --- | --- | --- | --- |
| TTO (days) | 4 (IQR: 1-12) | 5 (IQR: 2-10) | 6 (IQR: 3-12) | 5 (IQR: 3-9) | 1 (IQR:1-8) | 19 (IQR:5-694) | 5 (IQR: 2-11) | 8 (IQR: 2-75) | 4 (IQR: 2-11) |

TTO: time to onset

IQR: interquartile range

**Table S3.** **Clinical outcome proportions of ICANS with different CAR-T therapies**

|  |  | Tisagenlecleucel | Axicabtagene ciloleucel | Brexucabtagene autoleucel | Lisocabtagene maraleucel | Idecabtagene vicleucel | Ciltacabtagene autoleucel | Anti-CD19 CAR-T | Anti-BCMA CAR-T | Total CAR-Ts |
| --- | --- | --- | --- | --- | --- | --- | --- | --- | --- | --- |
| Proportion of death outcomes |  | 29.72% | 21.98% | 16.39% | 28.13% | 20.34% | 52.63% | 22.92% | 28.21% | 23.25% |
| Proportion of life-threatening outcomes |  | 9.64% | 8.65% | 6.56% | 9.38% | 5.08% | 0% | 8.56% | 3.85% | 8.27% |
| Proportion of hospitalization outcomes |  | 32.53% | 28.98% | 39.34% | 40.63% | 32.20% | 26.32% | 31.57% | 30.77% | 31.52% |
